# A program for sustained improvement in preventing ventilator associated pneumonia in an intensive care setting

**DOI:** 10.1186/1471-2334-12-234

**Published:** 2012-09-29

**Authors:** Raquel A Caserta, Alexandre R Marra, Marcelino S Durão, Cláudia Vallone Silva, Oscar Fernando Pavao dos Santos, Henrique Sutton de Sousa Neves, Michael B Edmond, Karina Tavares Timenetsky

**Affiliations:** 1 Intensive Care Unit, Hospital Israelita Albert Einstein, Av. Albert Einstein, 627/701 - 5º Andar – Bloco B, São Paulo, CEP 05651-901, Brazil; 2 Infection Control Unit, Hospital Israelita Albert Einstein, São Paulo, Brazil; 3 Medical Practice Department, Hospital Israelita Albert Einstein, São Paulo, Brazil; 4 Hospital Israelita Albert Einstein, São Paulo, Brazil; 5 Department of Internal Medicine, Virginia Commonwealth University School of Medicine, Richmond, Virginia, USA

**Keywords:** Ventilator associated pneumonia, Prevention, Intensive care, VAP bundle

## Abstract

**Background:**

Ventilator-associated pneumonia (VAP) is a common infection in the intensive care unit (ICU) and associated with a high mortality.

**Methods:**

A quasi-experimental study was conducted in a medical-surgical ICU. Multiple interventions to optimize VAP prevention were performed from October 2008 to December 2010. All of these processes, including the Institute for Healthcare Improvement’s (IHI) ventilator bundle plus oral decontamination with chlorhexidine and continuous aspiration of subglottic secretions (CASS), were adopted for patients undergoing mechanical ventilation.

**Results:**

We evaluated a total of 21,984 patient-days, and a total of 6,052 ventilator-days (ventilator utilization rate of 0.27). We found VAP rates of 1.3 and 2.0 per 1,000 ventilator days respectively in 2009 and 2010, achieving zero incidence of VAP several times during 12 months, whenever VAP bundle compliance was over 90%.

**Conclusion:**

These results suggest that it is possible to reduce VAP rates to near zero and sustain these rates, but it requires a complex process involving multiple performance measures and interventions that must be permanently monitored.

## Background

Ventilator-associated pneumonia (VAP) is a common infection in the ICU. [1] Recent studies describe a rate of 1 to 4 cases per 1,000 ventilator-days, although this can reach up to 10 cases per 1,000 cases ventilator-days in neonates and surgical patients [2,3]. The improvement in outcomes associated with recent initiatives suggest that many cases of VAP can be prevented by adhering to bundles of infection prevention measures [4,5].

The attributable mortality of VAP is around 4% to 9% varying with definitions, case-mix, causative microorganisms, and treatment adequacy [6,7]. VAP is also associated with considerable morbidity, due to increased length of hospital and ICU stay, prolonged mechanical ventilation and increased hospital expenses [8-10], as well as excessive utilization of antimicrobials with correspondingly higher costs [8,9].

As part of the 5 Million Lives campaign, endorsed by leading US agencies and professional societies, The Institute for Healthcare Improvement (IHI) recommends that all ICUs implement a ventilator bundle to reduce the incidence of VAP to zero [11]. Since 2007, we have implemented the VAP bundle in our ICU, including oral hygiene with 0.12% chlorhexidine and continuous aspiration of subglottic secretions (CASS) [4]. With these measures we were able to achieve zero incidence of VAP during a few months when a higher than 95% compliance rate with the VAP bundle was obtained [4]. However, to date there are no reports of sustained low incidence of VAP [4] (near zero).

The purpose of this quasi-experimental study was to evaluate whether the sustained implementation of the VAP bundle in our ICU could effectively reduce the incidence of ventilator-associated pneumonia (VAP).

## Methods

### Setting and study design

An interrupted time series study was conducted in a 38-bed medical-surgical intensive care unit (ICU) of a tertiary care, private hospital in São Paulo, Brazil. This is an open staffing model ICU where approximately 2,200 patients are admitted annually. This study was a quality improvement study that was approved by the Institutional Review Board of Hospital Israelita Albert Einstein (IRB). The requirement for informed consent was waived by our IRB in accordance with the Code of Federal Regulations and the Privacy Rule. This project was carried out after our previously published study from April 2007 to September 2008 [4]. Herein we report our observations for the period from October 2008 to December 2010 to evaluate whether the sustained implementation of the VAP bundle in our ICU could effectively reduce the incidence of VAP. All these hospital epidemiology data was analysed anonymously.

The VAP bundle included elevation of the head of the bed (HOB) (30–45 degrees); daily “sedation vacations” and assessment of readiness to extubate; peptic ulcer disease prophylaxis; and deep venous thrombosis/pulmonary thromboembolism (DVT/PE) prophylaxis for all ICU patients requiring mechanical ventilation. This ventilator bundle was monitored each weekday by an ICU nurse. She intervened in this process while performance monitoring was taking place at the bedside if non-compliance with an element of the bundle was detected (e.g., sedation was not stopped). We also intervened in other CDC evidence-based practices for prevention of ventilator associated pneumonia [3] including: 1) no routine changing of humidified ventilator circuits, 2) periodically draining and discarding condensate collecting in the ventilator tubing and, 3) changing the heat-and-moisture exchangers (HMEs) when they showed mechanical malfunction or became visibly soiled. These CDC process measures were audited twice yearly in a small sample of mechanically ventilated patients at random intervals.

Other interventions to control VAP in the ICU were implemented in October 2007 when oral decontamination with chlorhexidine 0.12% was introduced for all mechanically ventilated ICU patients [4]. In February 2008, the continuous aspiration of subglottic secretions (CASS) endotracheal tube was implemented for patients requiring mechanical ventilation and expected to require ventilation for longer than 24 hours [4].

**Figure 1:**
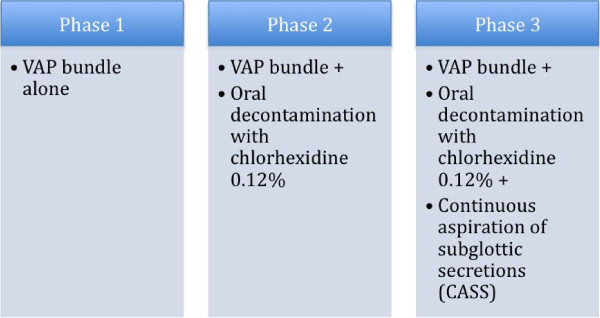
**Study design.** *Phases 1, 2 and 3 (from April 2007 to September 2008) are a consequence of our previous publication [4]. We extended data collection in this present manuscript (from October 2008 to December 2010) in phase 3.

Previously [4] we had compared the VAP bundle alone (phase 1), with the VAP bundle + oral decontamination with chlorhexidine 0.12% (phase 2), and the VAP bundle + oral decontamination with chlorhexidine 0.12% + continuous aspiration of subglottic secretions (CASS) endotracheal tube (phase 3). We then decided to analyze our performance after almost two years of all these interventions (including the VAP bundle) to determine whether this was a sustainable program for controlling ventilator associated pneumonia. We decided to extend data collection in phase 3 (VAP bundle + oral decontamination with chlorhexidine 0.12% + continuous aspiration of subglottic secretions (CASS) endotracheal tube) to evaluate this assumption (Figure 1). In summary, phases 1, 2 and 3 (from April 2007 to September 2008) are a consequence of our previous publication [4]. We extended data collection in this present manuscript (from October 2008 to December 2010) in phase 3. We provided monthly feedback on compliance with the bundle components to the ICU team (doctors, nurses and respiratory therapists). We also displayed posters in the ICU with bar charts showing compliance with the recommended procedures. These posters also showed the VAP rate as determined in surveys conducted by the Department of Infection Control and Hospital Epidemiology.

### Definitions

VAP surveillance was performed by trained infection control specialists using the US Center for Disease Control and Prevention/National Healthcare Safety Network (CDC/NHSN) definition [12] in an independence way from the treating ICU team, the incidence of VAP was expressed as cases of VAP per 1,000 ventilator-days and the incidence of ventilator associated tracheobronchitis (VAT) was expressed as cases of VAT per 1,000 ventilator-days.

VAP was defined as the sum of the clinical criteria as described the presence of fever (temperature >38°C), new or increased sputum production, in combination with radiologic evidence of a new or progressive pulmonary infiltrate, leukocytosis, a suggestive Gram’s stain, and grow of bacteria (not necessarily) in cultures of sputum, tracheal aspirate, pleural fluid, bronchoalveolar lavage (BAL), or blood [12]. Per the CDC/NHSN definition, microbiological data are not necessary for the diagnosis.

VAT was defined as the presence of fever (temperature >38°C), new or increased sputum production, a microbiologically positive respiratory sample, and the absence of pulmonary infiltrates on chest radiography.

### Microbiological methods

All isolates were identified by manual or automated methods and confirmed using the Vitek 2 system (bioMerieux Vitek, Inc., Hazelwood, MO).

### Statistical analysis

The variables of interest were those that indicated compliance with the VAP prevention measures. We used segmented regression analysis of interrupted time series [13] to assess the changes in VAP before and after implementation of the ventilator bundle, oral decontamination with chlorhexidine 0.12%, and CASS endotracheal tube for patients requiring mechanical ventilation, according to the interventional phases (Figure 1).

We adjusted a segmented regression model that allowed us to analyze a reduction (or an increase) in VAP rate at each study phase separately: (1) ventilator bundle only, April 2007 to October 2007; (2) ventilator bundle + chlorhexidine, November 2007 to February 2008; (3) ventilator bundle + chlorhexidine + CASS endotracheal tube, March 2008 to December 2010 (Figure 1).

The intercept and slope are the two parameters which define each segment of a time series. The intercept is the value of the series at the beginning of a given time interval, the slope is the change of the measure (VAP rate) over a certain period (e.g., a month). A change in slope (β) is defined by an increase or decrease in the slope of the time step after the intervention, compared with the time step preceding the intervention. It is important to mention that this is not the same as constructing three models of simple linear regression, because the third partition parameters depend on the previous partitions’ parameters.

All tests of statistical significance were 2-sided with a significance level set at 0.05. All the data analyses were performed using SPSS 16.0 and SAS 9.1; SAS Institute Inc, Cary, NC, USA.

## Results

### Compliance with process measures in each phase

In 2009, the process measures subject to analysis included 2,396 HOB elevation observations (98.6% compliance), 611 ventilator circuits without changes (99.8% compliance) and 611 observations of HMEs changes (95% compliance). Also included in the analysis were 2,396 daily sedation vacations, gastric prophylaxis opportunities and DVT/PE prevention opportunities with 98%, 99% and 98% compliance, respectively. There were also 611 observations of ventilator-circuit-tubing condensate with 92% compliance. CASS was performed in 342 patients since October 2008, and all patients requiring mechanical ventilation received oral decontamination with chlorhexidine 0.12% (Table 1).

**Table 1:** Characteristics of the sustained period of “getting to zero” VAP prevention program in the ICU

	**2009**	**2010**
**Patient-days (total)**	10,889	11,095
**Number of patients**	2,705	2,717
**Age, mean ± SD(in years)**	67±19	66±18
**Male, n (%)**	1,571 (58.1%)	1,587 (58.4%)
**APACHE, mean ± SD**	18±6	18±7
**Ventilator-days (total)**	3,009	3,043
**Ventilator utilization ratio**	0.28	0.27
**MV days – median(IQR)**	4 (1–22)	3.7 (1–23)
**Ventilator-free days**	7,880	8,052
**ICU LOS days –mean ± SD**	3.9±0.4	4.0±0.3
**Compliance with process measures,n (%)**		
HOB observations	2362/2396 (98.6%)	2260/2486 (90.9%)
Daily “sedation interruptions”	2358/2396 (98.4%)	2273/2486 (91.4%)
Gastric prophylaxis	2393/2396 (99.9%)	2276/2486 (91.5%)
DVT/PE prevention	2363/2396 (98.6%)	2266/2486 (91.1%)
Ventilator circuits without changes	610/611 (99.8%)	387/390 (99.2%)
HMEs changed	584/611 (95.5%)	368/390 (94.3%)
Ventilator-circuit-tubing condensate	564/611 (92.3%)	360/390 (92.3%)
**CASS endotracheal tube -n**	342	311
**Number of VAPs**	4	6
**Number of VATs**	3	6
**VAP rate per 1,000ventilator-days**	1.3	2.0
**VAT rate per 1,000ventilator-days**	1.0	2.0
**In-hospital mortality in VAPpatients, n (%)**	4/4 (100)	5/6 (83)
**In-hospital mortality in ICUpatients, n**	196	220
**In-hospital mortality in ICUpatients per 10,000 patientdays**	180	198

*CASS* Continuous Aspiration of Subglottic Secretions.

*DVT/PE* Deep Venous Thrombosis/Pulmonary Embolism.

*ICU LOS* Intensive Care – Length of Stay.

*HMEs* Heat-and-Moisture Exchanges.

*HOB* Head of the Bed.

*MV* Mechanical Ventilation.

*SD* Standard Deviation.

*VAP* Ventilator Associated Pneumonia.

*VAT* Ventilator Associated Tracheobronchitis.

In 2010, the analysis included 2,260 HOB elevation observations (91% compliance), 390 ventilator circuits without changes (99% compliance) and 390 observations of HMEs changes (94% compliance). Daily sedation vacations, gastric prophylaxis opportunities and DVT/PE prevention opportunities had 2,486 observations with 91% of compliance in all measurements (Table 1).

### Incidence density of VAP and in-hospital mortality of VAP patients

The incidence density of VAP per 1,000 ventilator days in the ICU was 1.3 in 2009 (10,889 patient-days) and in 2010 the incidence was 2 (11,095 patients-days). The incidence density of VAT per 1,000 ventilator days in the ICU was 1.0 (10,889 patient-days) in 2009 and in 2010 the incidence was 2 (11,095 patient-days).

Mechanical ventilation days, ICU length of stay, ventilator utilization ratio, number of VAPs, number of VATs, ventilator-days, ventilator-free days, in-hospital mortality of VAP patients and in-hospital mortality ICU patients are shown in Table 1.

**Figure 2:**
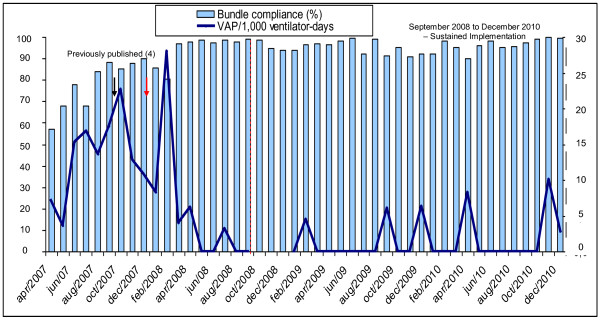
**Bundle compliance and VAP(ventilator associated pneumonia) ratefrom April 2007 toDecember 2010.** This chart shows extended data from the study published in AJIC 2009 (reference number 4). Oral decontamination with chlorhexidine 0.12% (since October/2007). Continuous aspiration of subglottic secretions (CASS) endotracheal tube (since February/2008).

Getting to zero VAP for one or more months has occurred since 2009 when there was greater than 95% compliance with the ventilator bundle, oral decontamination with chlorhexidine 0.12% and continuous aspiration of subglottic secretions (CASS) (Figure 2). In addition to this we continued to evaluate ventilator circuits without changes, HMEs changes and ventilator-circuit-tubing condensate.

**Figure 3:**
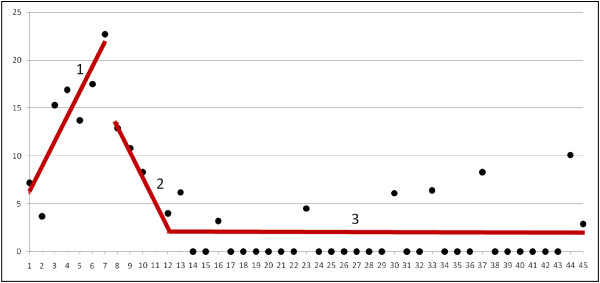
**Segmented regression of ventilatorassociated pneumonia (VAP) rateper 1,000 ventilator daysfrom April 2007 toDecember 2010.** Segmented 1: β10 = +6.08 p = 0.004; CI 95%: [(2.06 - 10.12)]. Segmented 1 (the slope): β11 = +2.59 p <0.001; CI 95%: [(1.47 - 3.71)]. Segmented 2: β20 = −11.24 p = 0.004; CI 95%: [(−18.60) - (−3.89)]. Segmented 2 (the slope): β21 = −2.30 p = 0.272; CI 95%: [(−6.48) - 1.88)]. Segmented 3: β30 = −2.67 p = 0.682; CI 95%: [(−15.83) - 10.47)]. Segmented 3 (the slope): β31 = +0.03 p = 0.610; CI 95%: [(−0.08) - 0.13)].

Segmented regression analysis (Figure 3) showed a statistically significant increase in VAP rate (β11 = +2.59; p < 0.001) in the first segment (ventilator bundle). The transition from the first segment (ventilator bundle) to the second segment (ventilator bundle + chlorhexidine) showed a significant decrease in VAP rate (β20 = −11.24; p < 0.001). The slope (β21) in the second segment was negative, indicating a reduction in VAP rate upon implementation of oral decontamination with chlorhexidine (β21 = −2.30 with p = 0.272). The transition from the second segment (ventilator bundle + chlorhexidine) to the third segment (ventilator bundle + chlorhexidine + CASS endotracheal tube) was not significant in VAP rate (β30 = −2.67 with p = 0.682). In the third segment the slope (β31) was practically zero, indicating that there was no reduction in VAP rate, which was maintained along the segment (β31 = 0.03 with p = 0.610).

### Microbiological features

As seen in Table 2, we had 10 cases of VAP, 4 in 2009 and 6 in 2010. Most patients were male (70%), with a median age of 58 years old (range 20 to 85 years), the median mechanical ventilation time was 9 days (range 5 to 33 days). Eighty percent of all the microorganisms identified were gram-negative, followed by viruses (10%), and 10% unidentified microorganisms.

**Table 2:** Characteristics of infections causing VAP during the sustained period of “getting to zero” VAP prevention program

**N**	**Year**	**Age**	**Gender**	**Diagnostic**	**MV time (days)**	**Respiratory specimen**	**Pathogen**	**Clinical outcome**
1	2009	85	Male	DLOC/Hyponatremia	16	Tracheal aspirate	*P.aeruginosa*	Death
2	2009	65	Female	Hypereosinophilia/ Myelopathy	5	BAL + Tracheal aspirate	*Acinetobacter baumannii*	Death
3	2009	20	Male	Correction of GERD	19	Tracheal aspirate	*P.aeruginosa*	Death
4	2009	23	Female	Liver failure/ liver transplant	5	Tracheal aspirate	*Acinetobacter lwoffii*	Death
5	2010	56	Male	Respiratory failure/ BCP	16	Tracheal aspirate	*P.aeruginosa*	Death
6	2010	62	Male	Carotid stenosis/ Endarterectomy	8	Tracheal aspirate	*S.marcescens*	Hospital discharge
7	2010	59	Male	Chagas cardiomyopathy	10	Tracheal aspirate	*E.cloacae*	Death
8	2010	55	Female	Hepatic encephalopathy	8	Nasopharyngeal swab	RSV	Death
9	2010	61	Male	Acute respiratory failure/ BCP	33	Tracheal aspirate	*K.pneumoniae* + *P.aeruginosa*	Death
10	2010	58	Male	Cranial trauma	7	Tracheal aspirate	*E.aerogenes* + *A.baumannii*	Death

*MV* Mechanical Ventilation.

*DLOC* Decreased Level Of Consciousness.

*GERD* Gastroenteral Reflux Disease.

*RSV* Respiratory Syncytial Virus.

*BCP* Bronchopneumonia.

*BAL* Bronchoalveolar lavage.

*Pseudomonas aeruginosa* accounted for over 40% of the gram-negative pathogens. The most prevalent pathogens overall were *Pseudomonas aeruginosa* and *Acinetobacter baumannii*. The majority of the pathogens were identified by tracheal aspiration. Only 25% of VAP patients (1/4) were investigate by BAL (Table 2).

The mortality rate was 100% for the patients with VAP in 2009 and 83.3% in 2010.

### Mechanical ventilation

**Figure 4:**
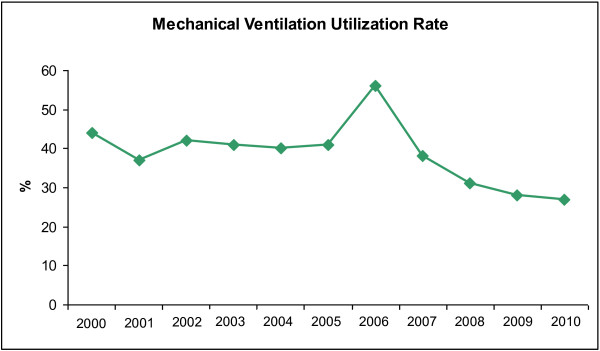
**Secular trends of mechanicalventilation utilization rate inICU**

The total time of mechanical ventilation was 6,052 days, with a utilization rate of 28% in 2009 and 27% in 2010 (Table 1). As seen in Figure 4, our mechanical ventilation utilization rates have been reduced since 2000, with a 17% drop from 2000 to 2010.

## Discussion

Since 2007, we have set as a priority in our hospital the eradication of nosocomial infections. To this end, we have developed a set of best practices for prevention of ventilator associated pneumonia (VAP). In order to achieve a reduction in VAP rates, we have applied the Institute for Healthcare Improvement bundle model and also implemented other preventive measures (oral chlorhexidine 0.12% and CASS endotracheal tube). Our VAP rates are discussed on a monthly basis at a multidisciplinary meeting with our hospital’s chief executive officer (CEO) and other senior management representatives responsible for ensuring that healthcare practices support a program for infection prevention and control that effectively prevents VAP.

Many hospitals have achieved the goal of getting VAP to zero [14,15], while others have managed to substantially reduce VAP rates, but believe that eliminating VAP in the intensive care unit may be an unrealistic goal [16]. In a previous publication [4], we have shown that this was only possible when the compliance with the VAP prevention bundle exceeded 95%, the CASS endotracheal tube was incorporated in daily practices and [17,18] oral hygiene with chlorhexidine was implemented [19]. In a recent systematic review and meta-analysis of patients at risk for ventilator-associated pneumonia, the use of endotracheal tubes with subglottic secretion drainage was shown to effectively prevent ventilator-associated pneumonia and to be possibly associated with reduced duration of mechanical ventilation and length of ICU stay [18]. We believe this might be the reason for our reduced time and utilization rate of mechanical ventilation, together with the daily sedation vacation included in the VAP bundle. We also believe that obtaining the commitment of all members of the ICU team was ultimately a factor in our success in the implementation of these procedures over the years.

**Figure 5:**
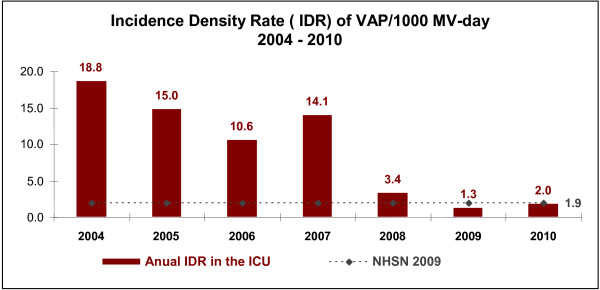
**Incidence density rate (IDR)of VAP/1000 ventilator-days from2004 to 2010 inthe ICU.**

Klompas et al. [20] have called attention to the problems that may arise when we use VAP as a quality indicator, including difficulties with the subjectivity implied by the current VAP definition. Moreover, Edmond has pointed out that the “getting to zero” approach may be associated with adverse unintended consequences [21]. Moreover, the CDC/NHSN definition has been shown to have lower sensitivity than the American College of Chest Physicians definition [22]. Even though we consider it important to report VAP rates, we believe we should continuously report our compliance to the prevention measures (VAP bundle) and the adverse events associated with mechanical ventilation in ICU patients [23]. However, we were able to show that the procedures implemented since 2007 have contributed to a significant reduction in our infection rates (Figure 5) and to a decrease in the use of mechanical ventilation in recent years (Figure 4). It is important to note that our VAP surveillance was performed by trained infection control practitioners using the US Centers for Disease Control and Prevention/National Healthcare Safety Network (CDC/NHSN) definition [12] throughout the study period. Even though we have failed to demonstrate a statistically significant result using the segmented regression analysis of VAP prevention, we have achieved a zero infection rate by applying all the VAP prevention measures recommended in the literature [8,9].

There are several limitations to this study. This is not a randomized trial but a quasi-experimental, interrupted time series study. Quasi-experimental study designs are frequently used when it is not logistically feasible to conduct a controlled trial. Thus, other unmeasured factors might have coincided with the interventions effective since April 2007 (implementation of the ventilator bundle), resulting in a decrease in VAP rates in our ICU. However, this seems unlikely because there had been no decrease in VAP rates over the previous several years (Figure 5). Data from our ICU in 2011 and in the first quarter of 2012 have shown that the VAP rate continues low (1.5 and 1.9, respectively). Finally, as this intervention was performed at a single medical center, it might be inappropriate to extrapolate our results (i.e. VAP mortality) to other hospitals. Even considering some aspects as the attributable mortality of VAP, other studies applying more sophisticated analysis such as multistate model that appropriately handle VAP as a time-dependent event or competing risk survival analysis have shown rates of attributable mortality as low as 10% [6,24]. Despite these limitations, our study further support the assumption that controlling VAP rates can be a sustained with the monitoring of multiple performance measures and quality improvement efforts.

## Conclusions

The process and the outcome measures for VAP presented here are derived from published guidelines and other relevant literature. While we recognize that the VAP definition may be subject to criticism due to its many subjective aspects, we managed to keep the whole team’s commitment to preventive measures for over two years, which demonstrates this is a sustainable program for preventing ventilator-associated pneumonia in the intensive care unit.

## Abbreviations

VAP: Ventilator-associated pneumonia; VAT: Ventilator associated tracheobronchitis; ICU: Intensive Care Unit; IHI: Institute for Healthcare Improvement; IRB: Institutional Review Board of Hospital Israelita Albert Einstein; NNISS: National Nosocomial Infectious Surveillance System; HOB: Elevation of the head of the bed; DVT/PE: Deep venous thrombosis/pulmonary thromboembolism; CDC/NHSN: Center for Disease Control and Prevention/National Healthcare Safety Network; CASS: Continuous aspiration of subglottic secretions endotracheal tube; HMEs: Heat-and-moisture exchangers; BAL: Bronchoalveolar lavage; CEO: Chief executive officer.

## Competing interests

The authors have declared that no competing interests exist.

## Authors’ contributions

RCE, MSD, CVS, KTT participated in the data collected and data analysis. AM, OFPS, MBE and KTT participated in the design and coordination. RCE, AM, CVS, HSSN, KTT and MBE helped to draft the manuscript and to provide critical review of the manuscript. All authors read and approved the final manuscript.

## Pre-publication history

The pre-publication history for this paper can be accessed here:

http://www.biomedcentral.com/1471-2334/12/234/prepub
